# The Reduction of the Combined Effects of Aflatoxin and Ochratoxin A in Piglet Livers and Kidneys by Dietary Antioxidants

**DOI:** 10.3390/toxins13090648

**Published:** 2021-09-13

**Authors:** Roua Gabriela Popescu, Sorin Avramescu, Daniela Eliza Marin, Ionelia Țăranu, Sergiu Emil Georgescu, Anca Dinischiotu

**Affiliations:** 1 Department of Biochemistry and Molecular Biology, Faculty of Biology, University of Bucharest, Splaiul Independentei, No. 91-95, 050095 Bucharest, Romania; roua.popescu@drd.unibuc.ro (R.G.P.); anca.dinischiotu@bio.unibuc.ro (A.D.); 2 Department of Organic Chemistry, Biochemistry and Catalysis, Faculty of Chemistry, University of Bucharest, Soseaua Panduri, No. 90-92, 050663 Bucharest, Romania; sorin.avramescu@g.unibuc.ro; 3 Laboratory of Animal Biology, National Institute for Research and Development for Biology and Animal Nutrition, Calea Bucuresti, No. 1, 077015 Balotesti, Romania; daniela.marin@ibna.ro (D.E.M.); ionelia.taranu@ibna.ro (I.Ț.)

**Keywords:** piglets, mycotoxins, CYPs protein expression, CYPs enzyme activity, feed additives, antioxidant effect

## Abstract

The purpose of this study was to investigate the combined effects of aflatoxin B1 and ochratoxin A on protein expression and catalytic activities of CYP1A2, CYP2E1, CYP3A29 and GSTA1 and the preventive effect of dietary byproduct antioxidants administration against these mycotoxin damage. Three experimental groups (E1, E2, E3) and one control group (C) of piglets after weaning (TOPIGS-40 hybrid) were fed with experimental diets for 30 days. A basal diet containing normal compound feed for starter piglets was used as a control treatment and free of mycotoxin. The experimental groups were fed as follows: E1—basal diet plus a mixture (1:1) of two byproducts (grapeseed and sea buckthorn meal), E2—the basal diet experimentally contaminated with mycotoxins (479 ppb OTA and 62ppb AFB1) and E3—basal diet containing 5% of the mixture (1:1) of grapeseed and sea buckthorn meal and contaminated with the mix of OTA and AFB1. After 4 weeks, the animals were slaughtered, and tissue samples were taken from liver and kidney in order to perform microsomal fraction isolation, followed by protein expression and enzymatic analyses. The protein expressions of CYP2E1 and CYP3A29 were up-regulated in an insignificant manner in liver, whereas in kidney, those of CYP1A2, CYP2E1 and CYP3A29 were down-regulated. The enzymatic activities of CYP1A2, CYP2E1 and CYP3A29 decreased in liver, in a significant manner, whereas in kidney, these increased significantly. The co-presence of the two mycotoxins and the mixture of grape seed and sea buckthorn meal generated a tendency to return to the control values, which suggest that grapeseed and sea buckthorn meal waste represent a promising source in counteracting the harmful effect of ochratoxin A and aflatoxin B.

## 1. Introduction

Mycotoxin contamination is a major concern with great impact on human and animal health [1], as mycotoxin may be tumorigenic, mutagenic, estrogen mimetic and immunosuppressive. They are absorbed through the gastrointestinal tract [2,3,4,5], distributed to different body parts and metabolized especially at the hepatic and renal level [6]. Humans and animals are exposed to these natural contaminants due to consumption of contaminated food and feed components, such as cereals, cereal products, fruits for direct consumption or their derived products [7,8]. Additionally, humans can be exposed to mycotoxin contaminated animal foodstuffs such as milk, eggs and meat, even fish meat [9,10]. One of the main difficulties encountered in controlling mycotoxins incidence and prevalence is that more than one type of mycotoxin is present in a batch of feed or cereal at the same time. Thus, ingestion of contaminated feed with several types of mycotoxins, even if they are at minimum concentrations, can cause numerous negative effects due to their additive, synergic or antagonist effects [11,12].

In general, the metabolism of xenobiotics, respectively the biotransformation process of toxic compounds, into compounds suitable for excretion depends on the structure and physical-chemical properties of parental compound and enzymes available in the exposed tissue [13,14]. Therefore, reactions of xenobiotic metabolism take place in three phases: phase I—modification, by adding a functional group, phase II—conjugation of the functional group with a compound in order to increase the hydrophily of the conjugate and phase III—excretion of the phase II metabolites [15]. As a result of these, changes in protein expression, ROS production and oxidative stress in affected cells or tissues [16,17,18,19] could occur. During the biotransformation process, beyond the increased hydrophilicity of xenobiotics, reactive intermediates could be formed, increasing toxicity [20].

Cytochrome P450s play an important role in Phase I of biotransformation of xenobiotics, especially those of the CYP1, CYP2, CYP3 and CYP4 families, which mainly catalyze the reactions of oxidation, reduction or hydrolysis [19]. Phase II metabolism involves glutathione *S*-transferase, sulfotransferases, *N*-acetyltransferases and uridine 5′-diphospho-(UDP)-glucuronosyltransferases [21,22,23]. In the end, Phase III transporters from liver, kidney and intestine remove the produced metabolites in the cells in an active manner. In pigs, such transporters involved in the elimination of mycotoxins’ metabolites conjugated with glucuronic acid, reduced glutathione and sulfate are represented by members of transporter family located in the basement membrane of polarized cells, such as MRP2, MRP4, Bcrp, Oat and Oct [19,23,24,25,26,27,28].

Studies regarding the mycotoxin toxicity in swine are numerous, but solutions for the reduction of these adverse effects are few. For example, the most used method to counteract the negative impact of mycotoxins in animals is adding “mycotoxin binders” or “mycotoxin modifiers”, which are very effective for aflatoxins [29] but have limited efficiency against other types of mycotoxins [30] and could bind also vitamins and trace elements [31], generating deficiencies. Adding different plant derived antioxidants in feed could be a better solution to diminish the deleterious effects of mycotoxins on animal health.

To our knowledge, studies regarding cytochromes P450 protein expressions corelated with enzyme activities have not been performed up to now. Previous studies demonstrated that for humans and pigs CYP1A protein expression of liver were decreased whereas CYP3A increased in mycotoxicosis [32,33]. Moreover, in pigs, after a 14-day exposure to T-2 mycotoxin, an inhibition of hepatic CYP3A activity was observed [34].

A number of studies have shown that plant compounds can modulate cytochrome P450s protein expressions and activities [35,36]. For example, in a group of pigs fed for 16 days with a basal diet supplemented with 10% dried chicory root, an important upregulation of CYP1A2 and CYP2A19 mRNA and a small increase in CYP2E1 mRNA increase was noticed, followed by a subsequent increase in the CYP1A2 and 2A19 protein expressions and activities [37]. Therefore, examining the tissue-specific patterns of cytochrome P450s protein expression levels and specific activities provides valuable information toward understanding mycotoxins metabolism and benefits of therapeutic compounds from plants to mitigate the harmful damage produced by the co-presence of mycotoxin in feed.

The aim of our study was to investigate the combined effects of aflatoxin B1 and ochratoxin A in the piglet’s liver and kidney on protein expression and catalytic activities of CYP1A2, CYP2E1, CYP3A29 and GSTA1 and the preventive effect of dietary byproduct antioxidants administration against these mycotoxins. 

## 2. Results

### 2.1. Relative Protein Expression

In liver, the relative protein expression for CYP1A2 increased by 58% in the E1 group, fed with a basal diet supplemented with a mixture of grape seeds and sea buckthorn meal and decreased by 7% for the E2 group. The addition of a mixture of grape seed and sea buckthorn meal to weaned piglets diet contaminated with the mix of AFB1 and OTA (E3 group) has determined an upregulation by 30% of the expression of CYP1A2, compared to control group. At renal level, the CYP1A2 relative protein expression decreased by 18% for the E1 group and 36% for the E2 group, respectively, and increased by 28% for the E3 group compared to control one. These results were basically consistent with our previous data regarding to the relative mRNA expression for *CYP1A2* [38].

**Figure 1:**
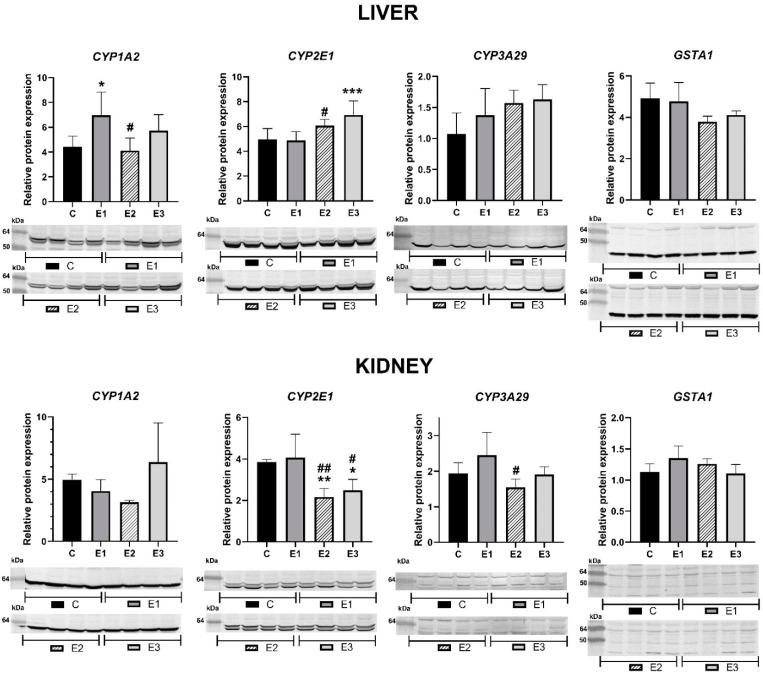
Relative protein expression and the corresponding quantification of Western blot images for CYP1A2, CYP2E1, CYP3A4 and GSTA1 in the hepatic and renal microsomal fractions of weaned piglets subjected to experimental diets. Calnexin band (70 kDa) was used as reference protein. The control group (C) were fed a basal diet. The experimental groups were fed as follows: the basal diet plus a mixture (1:1) of two byproducts (grapeseed and sea buckthorn meal) (E1 group), the basal diet artificially contaminated with AFB1 and OTA (E2 group), and the basal diet containing the mixture (1:1) of grapeseed and sea buckthorn meal and contaminated with the mix of AFB1 and OTA (E3 group). The data are illustrated as average values of the groups (*n* = 4) ± standard deviation of the mean (SE) and statistical significance related to the control group level. * E1/E2/E3 vs. C; # E2/E3 vs. E1; *, # *p* < 0.05; **, ## *p* < 0.01; *** *p* < 0.001.

As for the protein expression of CYP2E1 in liver, it was practically unchanged in E1 group, and an up-regulation of 22% in E2 group and a significant one of 40% in E3 group (*p* < 0.001) compared to the control group were noticed. In contrast, in the kidney samples, protein expression of CYP2E1 decreased in E2 (*p* < 0.01) and E3 (*p* < 0.05) groups compared to liver ones. In the case of group E3, it was increased by 15% compared to the E2 group and significantly decreased by 39% compared to the E1 group (Figure 1), while for the E2 group, CYP2E1 expression level decreased significantly by 35%, compared to the control one.

For CYP3A29 protein expression, in the case of liver, administration of the basal diet enriched with a mixture of grape seed and sea buckthorn meal (group E1) increased the relative protein expression by 29% for the E1 group, by 47% for the E2 group and by 52% for the E3 group, compared to the control group level. Relative protein expression for CYP3A29 in the kidney increased by 27% for the E1 group and decreased by 20% for E2 and by 1.6% for E3 compared to the control group (Figure 1).

Interestingly, in liver samples, GSTA1 relative protein expression showed a decrease of 3.2% for the E1 group, 23% for the E2 group and 17% for the E3 group, compared to the control group. In kidney, the relative protein expression for GSTA1 showed increases of 19% and 11% for the E1 and E2 groups, respectively, and a decrease of 2.5% for the E3 group compared to the control one.

### 2.2. Enzymatic Activities

In liver, introduction in the diet of a mixture of grape seed and sea buckthorn meal diminished the activities of CYP1A2, CYP3A29 and GSTA1 in a significant (CYP1A2 and CYP3A29) or insignificant way (GSTA1), whereas CYP2E1 one increased insignificantly. The presence of OTA and AFB1 diminished significantly the CYP1A2, CYP2E1 and CYP3A29 specific activities and insignificantly the GSTA1 one. The concomitant administration of mixture of grape seed and sea buckthorn meal and AFB1 and OTA generated a decrease of all CYPs specific activities except the GSTA1 one compared to control.

In kidney, the mixture of grape seed and sea buckthorn meal added to feed determined an increase of enzymatic activities of CYP1A2, CYP2E1 and GSTA1 and a very significant decrease of CYP3A29 one compared to control (*p* < 0.001). The presence of AFB1 and OTA in piglets’ feed increased all four enzymatic activities, whereas the co-presence of the two mycotoxins and the mixture of grape seed and sea buckthorn meal generated a tendency to decrease in all enzymatic activities toward the control values.

**Figure 2:**
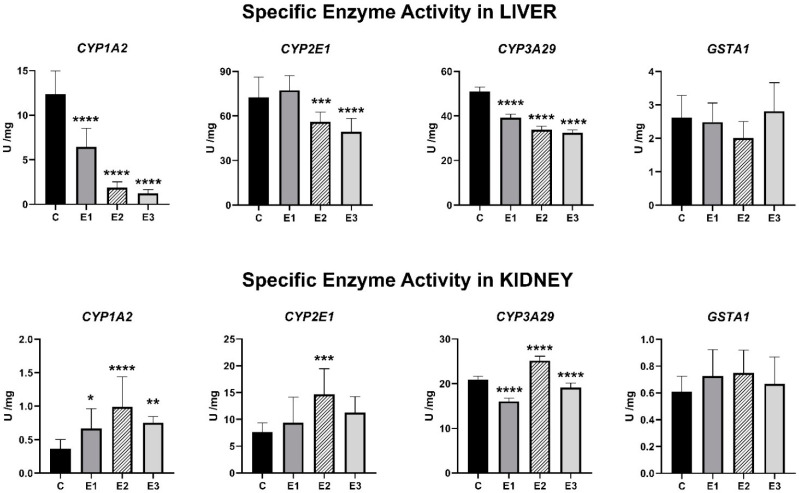
Enzymatic specific activity in the hepatic and renal microsomal fractions for CYP1A2, CYP2E1, CYP3A29 and GSTA1 of weaned piglets subjected to experimental diets. The control group (C) were fed a basal diet. The experimental groups were fed as follows: the basal diet plus a mixture (1:1) of two byproducts (grapeseed and sea buckthorn meal) (E1 group), the basal diet artificially contaminated with AFB1 and OTA (E2 group) and the basal diet containing the mixture (1:1) of grapeseed and sea buckthorn meal and contaminated with the mix of AFB1 and OTA (E3 group). The data are illustrated as average values of the groups (*n* = 4) ± standard deviation of the mean (SE) and statistical significance related to the control group level. * *p* < 0.05; ** *p* < 0.01; *** *p* < 0.001; **** *p* < 0.0001.

The hepatic CYP1A2 activity decreased significantly (*p* < 0.001) by 85% for the E2 group (1.88 U/mg) compared to control (12.35 U/mg) (Figure 2). In the kidney samples, CYP1A2 activity increased significantly (*p* < 0.001) by 2.7 times in the case of the E2 group (0.98 U/mg) compared to the control one (0.36 U/mg).

The specific activity of hepatic CYP2E1 decreased by 27% and 36% for the E2 group and E3 group (*p* < 0.001), respectively, compared to the E1 group. In contrast, the administration of basal diet supplemented with a mixture of AFB1 and OTA resulted in an increase of 56% in the specific activity of renal CYP2E1 compared to the E1 group level.

The administration of basal diet enriched with a mixture of grape seed and sea buckthorn meal (E1 group) increased significantly (*p* < 0.001) the CYP3A29 specific activity in the liver by 15% compared to the E2 group level. Another contrast was observed in the renal CYP3A29 specific activity with a significant decrease of 57% (*p* < 0.001) in the E1 group and 31% in the E3 group (*p* < 0.001), compared to the E2 group level (Figure 2).

In the case of hepatic GSTA1, the specific activity showed decreases of 6% and 23% for the E1 and E2 groups, respectively, and an increase of 7% in the E3 group, compared to the control level. The renal GSTA1 specific activity showed an increase in experimental groups, compared to the control level.

Analyzing Figure 2, it can be noticed that mixture of grape seed and sea buckthorn meal decreased the CYP1A2 and CYP3A29 specific activities at hepatic level in a significant way, whereas at renal level, the CYP1A2 specific activity was significantly increased (*p* < 0.01). Moreover, the presence of OTA and AFB1 in piglet’s feed decreased significantly the hepatic CYP1A2, CYP2E1 and CYP3A29 specific activities (*p* < 0.001), whereas in the kidney, the CYP1A2, CYP2E1 and CYP3A29 specific activities were increased significantly (*p* < 0.01). The concomitant administration of the mixture of grape seed and sea buckthorn meal and OTA and AFB1 determined the restauration of specific activity levels to control ones only in the kidney samples.

## 3. Discussion

Pigs are important non- rodent models in toxicology as well as in biomedical research [39] due to the fact that they have genetic and physiological traits similar to humans [40]. Pigs’ liver presents the highest constitutive protein expressions and enzymatic activities of CYP1A1, CYP 1A2, CYP2E1 and CYP3A compared to other organs such as muscle, adipose tissue and intestine. Their kidney is also metabolically active. The metabolizing enzymes are primarily CYPs and are presented at the highest level in the renal proximal convoluted tubules. In pigs, they have not been studied extensively [39].

The CYP450 enzyme expressions and activities can be regulated by many different factors, including genetic polymorphism, epigenetic influences on xenobiotic metabolism, non-genetic host factors and depend on gene expression, mRNA translation, post-translational processes, protein expression level and inhibition or activation process of the catalytic activity of enzymes [41].

Regulation of porcine CYP450s expression in renal tissue has received less attention. In our study, changes in the renal tissue, compared to the liver samples in the expression level of cytochromes P450 can be observed.

The use of phenolic antioxidants as a feed supplement to pigs has recently attracted considerable attention because of their positive impact on meat quality, particularly by the reduction of skatole levels, which together with androsterone contributes to the development of boar taint [42]. However, there are a number of bioactive secondary metabolites in vegetal by-products, such as sesquiterpene lactones [43], one of these being artemisinin (a sesquiterpene lactone from *Artemisia* sp.) that has been shown to up-regulate CYP3A4 and CYP2B6 expression in humans and mice by binding to the nuclear receptors PXR and CAR [44]. A study of our group has demonstrated that feeding grapeseed and sea buckthorn meal, containing ferulic acid, p-coumaric acid, caffeic acid, vanillic acid, luteolin, quercetin, rutin, epicatechin, catechin in the diet of OTA and AFB1-intoxicated pigs decreased the *CYP1A2*, *CYP1A19*, *CYP2E1*, *CYP3A29* and *CYP4A24* gene expression, suggesting the decrease of bioactivation of these mycotoxins, probably resulting in a diminished toxicity in both organs, as the histological studies have revealed [38].

Previously, direct effects of phytochemicals on CYP450 dependent activity have been shown [45,46,47]. Scott et al. [48] demonstrated that CYP3A4, CYP19 and CYP2C19 activities in vitro could be modified by various plant constituents, and the magnitude of inhibition is dependent on concentration of the bioactive constituents in extract. Thus, to further investigate the impact of grapeseed and sea buckthorn meal on CYP450s activity, the direct effect of by-products in the diet of pigs was investigated.

In humans, CYP1A2 enzyme plays an important role in the metabolism of several clinically used drugs. It is one of the major P450 enzymes and accounts for approximately 13% of the total content of this enzyme group in the human liver [49]. CYP1A2 mRNA content shows an up to 40-fold variability between individuals [50] and corresponding variability of enzyme activity and drug metabolism [51,52]. The genetic variation in CYP1A2 activity is estimated to be up to 75% depending on environmental factors [53]. According to Klein et al. [54], the genetic variation of CYP1A2 activity might only account for 42%, 38% and 33% of the catalytic activity, protein expression and mRNA levels, respectively, in human liver samples. Taking into account the predominant role of CYP1A2 in activation of toxic xenobiotics compared to its metabolism of prescription drugs, there are many epidemiological reports examining the role of CYP1A2 variants, metabolism of procarcinogens and cancer risk.

In liver, both AFB1 and OTA are metabolized in reactions catalyzed by CYP1A2 and CYP3A4 [55,56]. AFB1 metabolization requires oxidation of the 8,9 double bond to yield the biologically active AFB1-8,9-epoxide that can react with DNA. At high concentrations of AFB1, the major producer of this metabolite is CYP3A4 [55], whereas at lower concentrations, the main enzymatic player is CYP1A2 [57]. Recent studies revealed that, a high dose of OTA, i.e., 3 mg per kg body weight given to ICR-type mice, diminished the protein expression of CYP1A2 [56]. This could be the reason for which CYP1A2 protein expression decreased slightly in the E2 group compared to the control one. AFB1 is also metabolized into a number of hydroxylation products, such as aflatoxin Q1 (AFQ1), aflatoxin P1 (AFP1), aflatoxin B2a (AFB2a), aflatoxin M1 (AFM1), aflatoxicol (AFL) and aflatoxicol H1 (AFH1). These could exert an inhibition of CYP1A2 activity as previously it was proved for other natural compounds [58]. The lower protein expression together with the inhibitory action of hydroxylated compounds could cooperate for the decrease of CYP1A2 specific activity in E2 group. The addition of several flavonoids and phenolic acids present in the byproducts mixed in feed [38] could explain the lower specific activity of CYP1A2 in the liver of the E3 group.

Taking into account that the kidney is implicated in the removal of metabolic wastes and xenobiotics from the circulatory system, a relatively high level of toxic substances can be formed during the urine concentration process [59]. In our experiment, probably the concentration of AFB1 and OTA increased in kidney and induced CYP1A2 biosynthesis. This enzyme catalyzes the oxidation of the xenobiotics and generates superoxide and hydrogen peroxide. Recent data revealed that exposure to OTA and AFB1 increased ROS level in HK-2 human proximal tubule epithelial cells [59] in chickens’ kidneys [60]. If these overwhelmed the capacity of antioxidant system [61], hydrogen peroxide could accumulate and operate as a negative feedback loop for CYP1A transcription [62]. On the other hand, advanced oxidation protein products that formed due to the ROS attack on proteins down-regulated the expression of CYP1A2 and CYP3A4 in the kidneys of rats’ models for chronic kidney disease [63]. Moreover, probably, antioxidants present in the by-products diminished ROS level, and the protein expression of CYP1A2 was up-regulated in the kidneys of E3 group individuals.

In our opinion, in the kidneys of the E1 group, the increase of CYP1A2 activity might be due to the influence of oleic and linoleic acids, beyond other unsaturated fatty acids and polyphenols present in the byproducts added to piglets’ feed [38]. Taking in account that CYP1A2 is located in the endoplasmic reticulum (ER) membrane, its activity could be dependent on this membrane’s fluidity. The ER membrane contains high quantities of phosphatidylcholines, phosphatidylethanolamines and phosphatidylinositol and low ones of sphingolipids and cholesterol, and as a result, the lipid packing is tight and ordered [64]. The *cis*-unsaturated fatty acids such as oleic and linoleic ones supplied by diet could be used for de novo synthesis of these phospholipids in ER, and once existing, could decrease the packing compactness of acyl chains, rising membrane fluidity [65]. Furthermore, flavonoids and iso-flavonoids might enter the hydrophobic core of membrane, causing an important decrease of lipid fluidity [66].

The active site of CYPs is present on the cytosolic side of ER membrane, is buried in the enzyme structure and contains the hem cofactor. It could adopt several conformations, and the substrates would bind to its most suitable conformation [67]. The increased fluidity of ER membrane could facilitate adopting such a suitable conformation, and the catalytic activity would be increased in E3 kidneys compared to control. For the E2 group, the high concentration of AFB1 and OTA due to the urine concentration process could increase specific activities of CYP1A2 and CYP3A4, compared to the control one. Moreover, it appears that the co-administration of by-products and mycotoxins (E3 group) decreased these two specific activities compared to those of the E2 group, still remaining higher in comparison with E1 and control groups.

CYP2E1 is an enzyme responsible for the metabolism of a large number of xenobiotics, such as aliphatic, aromatic and halogenated hydrocarbons, many of which are solvents and industrial monomers, mycotoxins and other drugs [68,69]. CYP2E1 is localized in the centrilobular region of the liver, but has also been detected in lung, bronchial tissue, kidneys, nasal mucosa, intestine and lymphocytes [70]. The regulation of CYP2E1 expression depends on transcriptional, post-transcriptional and post-translational factors. Increased hepatic CYP2E1 protein expression for the E2 group might be due to mycotoxins binding to CYP2E1 that stabilize the protein and thus increase CYP2E1 content [70,71]. As shown in Figure 1, renal CYP2E1 protein expression was down-regulated in E2 group. However, inclusion of by-products in mycotoxins contaminated diet effectively restored this decrease in E3 group.

In contrast, the decrease in enzyme activity in liver could be due to poorer transcription or stability of *CYP2E1* gene product rather than a functional change in the enzyme [72], which is in agreement with those reported in our previous study by Popescu et al. [38], when comparing *CYP2E1* mRNA levels to the levels of enzyme activities found for all experimental groups. Moreover, in the liver of E2 group individuals, possibly as response to the increased oxidative stress caused by induction of CYP2E1 activity in hepatocytes, glutathione S-transferase activity was found to be up-regulated, in agreement with the study of Mari and Cederbaum, [73]. The catalytic activity of CYP2E1 has been associated with susceptibility to toxicity under industrial exposure to chemicals such as benzene [68]. Therefore, probably, in the present study, renal CYP2E1 activity increased for the E2 group and was restored for the E3 group, compared to the control level.

It should be noted that our study did not fully cover the entire CYP450s enzyme families with respect to mRNA expression and that this may partially explain the discrepancies between mRNA expression, protein expression and activity measurements. The discrepancies between mRNA expression and activity between experimental groups and the contradictory results on CYP2E1 need to be addressed in further studies.

In general, the studies regarding porcine CYP450s enzymes have focused on the impact of xenobiotics or antioxidant compounds only at molecular level and for a single type of tissue [38,74,75,76,77,78], while investigations regarding correlation between enzyme activity, protein level and mRNA transcript are few.

The pig is a relevant animal model for xenobiotics metabolic studies due to similarity to humans [79], therefore, studies on porcine CYP3A29 are important for a better understanding of mycotoxins co-exposure in vivo and metabolism studies [80].

Previous works have demonstrated that CYP3A constitutive expression is regulated by nuclear transcription factor Y; specificity protein 1 [81,82]; hepatocyte nuclear factors 1α, 3γ and 4 [83,84,85]; upstream stimulatory factor 1 [86]; activator protein 1 [87] and CCAAT/enhancer-binding proteins α and β [85,86]. In the presence of xenobiotics, induced expression of CYP3A is mediated by Pregnane X Receptor [80], constitutive androstane receptor (CAR), glucocorticoid receptor (GR) and vitamin D receptor (VDR) [81,88,89].

We demonstrated that by-products addition increased CYP3A29 protein expression in liver and kidney, while CYP3A29 activity was decreased, and only in kidneys, the tendency was opposite in the case of AFB1 and OTA co-contamination of the feed. The restoration of CYP3A29 relative protein expression level in kidney for the E3 group compared to the control one showed that the addition of a mixture of grapeseed and sea buckthorn meal by-products in mycotoxins contaminated diets favored the elimination processes and generated an adaptive response to the perturbation of hepatic and renal metabolism. The results for renal CYP3A29 activity were according with those reported in our previous study by Popescu et al. [38], when comparing *CYP3A29* mRNA levels to the levels of enzyme activities found for the E1 and E2 groups. Although, correlation of enzymatic activity from transcriptomic data was observed in a study [90], based on our results, we would argue that the differences between protein expression and specific activity are due to protein ability to bind specifically substrates and the way in which the enzyme is regulated [91].

Phase-I enzymes catalyze the primary reactions of xenobiotic detoxification [92]. Due to their electrophilic nature, phase-I metabolites have a potential to form stable adducts with nucleic acids and proteins, which act as cell-toxic and carcinogenic compounds [93,94]. The cytotoxic intermediates metabolites generated from Phase I are conjugated with hydrophilic moieties to form more readily excreted metabolites [95] by phase II enzymes, such as UDP–glucuronosyltransferases (UGTs), sulfotransferases, glutathione S-transferases, *N*–acetyltransferases, *N*–methyltransferases, phenol and catechol *O*–methyltransferase, Thiol methyltransferase and amino acid *N*–acyltransferase [96,97].

Glutathione *S*-transferases (GSTs) are dimeric enzymes (EC 2.5.1.18) that catalyze the conjugation of the reduced form of glutathione (GSH) to a broad variety of xenobiotic substrates including arene oxides, mycotoxins, lipoperoxidation-derived aldehydes, highly reactive aldehydes and other substrates [97,98,99]. GSTA1 is a cytosolic isoenzyme containing 222 amino acids from class alpha (A), based on amino acid sequence and substrate specificity of GSTs, with expression in liver and kidney. It is encoded by *GSTA1* gene [100]. The mycotoxins present in the feed and food chain such as AFB1 that are converted to AFB1-8,9-exo-epoxide (AFBO) via P450 metabolism are substrates for GSTs [101]. The ability of AFBO to conjugate with GSH reflects the expected sensitivity to AFB1-induced carcinogenesis since the pig is prone to develop hepatic tumors in vivo in the presence of xenobiotics [102,103].

Due to the lack of data about GSTs protein expression and catalytic activity in hepatic and renal tissue of pigs, we focused in our study on GSTA1 expression level. In the present study, the variation of GSTA1 protein expression and specific activity were decreased in liver and increased in kidney for the E2 group and restored for the E3 group, compared to the control level. The difference between liver and kidney could possibly be due to the physiological features of these pigs’ organs [101], which correlates with the antagonist actions by addition of phenolic antioxidants [104] such as a mixture of grapeseed and sea buckthorn meal by-products (E1 group). The restoration of GSTA1 protein expressions and specific activities post-addition of phenolic antioxidants could be due to the counteracting effect on reactive oxygen species generated by mycotoxins metabolism that might decrease the GSH content [104]. Moreover, recent studies revealed that, in rats, phase I metabolites from OTA react with GSH to produce GSH-conjugates. In kidney, OTB-GSH is the major metabolite, and therefore, higher levels of GSH conjugates suggest a greater level of OTA bioactivation and greater sensitivity of the kidney to OTA [105,106]. Moreover, Gekle et al. [107] demonstrated that kidney is the target organ of OTA toxicity, probably because this mycotoxin is actively accumulated in kidney cells, due to unfavorable kinetics of renal elimination [106].

## 4. Conclusions

As far as we know, this is the first study analyzing the protein expressions of CYP1A2, CYP2E1, CYP3A29 and GSTA1 in comparison with their specific enzymatic activities in piglets’ livers and kidney under combined exposure to AFB1 and OTA. A tissue-dependent response was noticed. Taking in account that CYP1A2, CYP2E1 and CYP3A29 activities were raised in the kidney of the E2 group individuals and decreased in those of the E3 group, it appears that the kidney was more affected compared to liver, and addition of by-products in the piglets’ feed was beneficial. These findings along with those of gene expression suggest that grapeseed and sea buckthorn meal waste represent a promising source for counteracting the harmful effect of ochratoxin A and aflatoxin B. The discrepancies between protein expression and activity between experimental groups and the mechanisms involved need to be addressed in further studies.

## 5. Materials and Methods

### 5.1. Animals, Treatment and Sampling

Three experimental groups (E1, E2, E3) and one control group (C) of piglets after weaning (TOPIGS-40 hybrid, *n* = 10 per group, housed in pen two replicates of 5 pigs per pen) with an average body weight of 9.11 ± 0.03 kg were fed with experimental diets for 30 days. They fed on a basal diet which was served as a control (control group—C) with normal compound feed for starter piglets (corn 68.46%, soya meal 19%, corn gluten 4%, milk replacer 5%, L-lysine 0.3%, DL-methionine 0.1%, limestone 1.57%, monocalcium phosphate 0.35%, salt 0.1%, choline premixes 0.1% and 1% vitamin-mineral premixes). The feeding treatments for the experimental groups were as follows: group E1 received basal diet including a mixture (1:1) of two meal by-products (grape seed and sea buckthorn) in a percentage of 5% which replace corn and soya bean meal; group E2 received the basal diet artificially contaminated with a mixture of 62 ppb aflatoxin B1- AFB1 and 479 ppb ochratoxin A-OTA (E2 group) and group E3 get the basal diet with 5% by-product meal mixture and contaminated with AFB1 and OTA mycotoxins (62 and 479 ppb respectively). The AFB1 and OTA contaminated material was kindly provided by Dr. Boudra and Dr. Morgavi (I.N.R.A. Clermont Ferrand, Clermont-Ferrand, France) and was produced by the cultivation of Aspergillus flavus and Aspergillus ochraceus, respectively, on wheat as already described by Boudra et al. [108] resulting in a AFB1 concentration of 30 mg/kg and OTA 230 mg/kg. The grape seed meal and sea buckthorn meal were provided by two local commercials S.C. OLEOMET-S.R.L. and BIOCATINA, Bucharest, Romania. The mixture of mycotoxins was kindly provided by I.N.R.A, Centre of Clermont Ferrand. Data regarding diet composition, fatty acid composition of grapeseed and sea buckthorn, flavonoids and phenolic acids composition of byproducts, mineral composition of byproducts, animal performance and biomarkers of liver and kidney function in plasma were published previously by Popescu et al. 2021. Assigned diet and water were provided ad libitum during the experiment. At the end of the experiment (day 30), animals were slaughtered with the approval of the Ethical Committee of the National Research-Development Institute for Animal Nutrition and Biology, Balotești, Romania (Ethical Committee no. 118/2 December 2019) and in accordance with the Romanian Law 206/2004 and the EU Council Directive 98/58/EC for handling and protection of animals used for experimental purposes. From four animals per group, the liver and kidney were collected and perfused with ice-cold saline solution to remove blood. Right liver lobe and renal cortex samples were collected on ice from all animals and were stored at −80 °C until the microsomal fraction isolation.

### 5.2. Isolation of the Microsome Fraction

The microsome fraction was isolated according to Rasmussen et al., [37], with slight modifications. Briefly, 6 g liver/kidney tissue were minced with sharp scissors and placed into a pre-chilled Dounce glass tube and added 4–5 volumes (4–5 mL of buffer per g of tissue) of ice-cold Tris–sucrose buffer (10 mM Tris–HCl, 250 mM sucrose, pH 7.4) and homogenized on ice using a thigh-fitting Teflon pestle attached to a Glas-Col Tissue Homogenizing System (Cole-Parmer, setting 70) for 3 min with a 30 s break at each 1 min. After 10 min centrifugation at 10,000× *g*, 4 °C, the supernatant (crude homogenate) was used for microsome isolation. Therefore, crude homogenate was diluted in Tris–sucrose buffer to a final volume of approximately 18 mL and centrifuged (Beckman Coulter Optima™L-80 XP Ultracentrifuge) with a fixed-angle rotor (Beckman 90 Ti) in OptiSeal tubes (Beckman, Ref 361623) at 100,000× *g* for 60 min at 4 °C. After ultracentrifugation, the supernatant obtained was collected as cytosolic fractions and the microsomal pellet were suspended in a buffer containing 50 mM Tris–HCl, 10 mM KH_2_PO_4_, 0.1 mM EDTA, 20% glycerol (pH 7.4) and stored at −80 °C in aliquots of 200 μL for later western blot and enzymatic assays. The purity of the microsomal and cytosolic fractions obtained after ultracentrifugation was assessed by immunoblotting for CYP1A2, CYP2E1, CYP3A29 and GSTA1. All steps were carried out on ice.

### 5.3. Western Blot Analysis

The obtained microsomes were used to evaluate protein expression for CYP1A2. CYP2E1, CYP3A29 and GSTA1 with Western blotting technique. Quantities of 30 μg microsomal protein from each sample were denatured by heating in the presence of a 5× Laemmlli buffer for 5 min at 95 °C. After cooling, the denatured proteins samples were separated by sodium dodecyl sulphate-polyacrylamide gel electrophoresis (SDS-PAGE, 10% separating gel) under reducing conditions in TRIS-glycine-SDS buffer at 90 V for 2 h. Proteins were transferred onto 0.4 μm poly-(vinylidene difluoride) membrane (Millipore, Billerica, MA, USA) in a wet transfer system (Bio-Rad, Hercules, CA, USA). Membrane blocking, and the incubations of primary and secondary antibodies were performed using the Western Breeze Chromogenic kit (Invitrogen, Themo Fischer Scientific, Waltham, MA, USA), and the membranes were processed according to manufacturer’s instructions. Primary antibodies used were rabbit polyclonal antibodies anti-CYP1A2 (MyBioSource, San Diego, CA, USA, MBS9605022, 1:750), anti-CYP2E1 (MyBioSource, San Diego, CA, USA, MBS9605034, 1:750), anti-Cytochrome P450 Enzyme CYP3A4 (Merck, Temecula, CA, USA, AB1254, 1:1000) and anti-GSTA1 (NovusBiologicals, NBP1-33586, Centennial, CO, USA, 1:1000). The obtained bands were visualized with the ChemiDoc MP system (Bio-Rad, Hercules, CA, USA) and quantified using ImageLab software 5.1 (Bio-Rad, Hercules, CA, USA). Each sample analyzed was normalized to the expression corresponding to the calnexin band (Merck, Temecula, CA, USA, AB2301, 1:750) used as a control of protein loading.

### 5.4. Enzymatic Activity Assays

In accordance with protein expression evaluation from microsome fraction, enzymatic activity was also evaluated for: CYP1A2, CYP2E1, CYP3A29 and GSTA1.

#### 5.4.1. CYP1A2

The activity of CYP1A2 was measured according to Hanioka et al. [109], with modifications. Therefore, a quantity of 200 μg microsomal protein was preincubated with 2 μM methoxy resorufin (MROD) (Sigma, Saint Louis, MO, USA, M1544-1MG) in 50 mM K_2_HPO_4_/KH_2_PO_4_ buffer (pH 7.4) in a final volume of 425 μL at 37 °C for 1 min. After preincubation, the reaction was started by adding 75 μL of 6.666 mM NADPH (final concentration of NADPH: 1 mM), incubated at 37 °C for 5 min and stopped with 500 μL of 100% ice-cold methanol with vortexing. After cooling on ice for 5 min, the samples were centrifuged at 10,000× *g* for 10 min at 4 °C, and the supernatant was collected and added into amber vials for HPLC analysis. The production of resorufin was analyzed by a HPLC method according to Wanwimolruk et al. [110] using a Varian HPLC system, Prostar 410 solvent delivery pumps, Prostar 350 autosampler, and column oven, equipped with a Pack Pro C18 150 × 4.6 mm I.D. S-3 μm, 12 nm column (YMC). The column was kept at 40 °C. A volume of 20 μL of sample was injected, and the metabolite resorufin was eluted isocratically with 20 mM K_2_HPO_4_/KH_2_PO_4_ buffer (pH 6.8), methanol and acetonitrile (30:35:35 *v*/*v*) at a flow rate of 0.5 mL/min (Supplementary Materials Figure S1B). The eluent was monitored by a fluorescence detector ProStar 363 with excitation and emission wavelengths of 560 and 586 nm, respectively. The obtained data were analyzed using Varian Workstation 6.3 software (Santa Clara, CA, USA). For quantification of resorufin, a standard calibration curve of resorufin (Sigma, Saint Louis, MO, USA, 73144-20MG) from 1 to 200 nM was prepared (Supplementary Materials Figure S1A). The enzyme activity was calculated by relating the amount of produced resorufin to blank incubations and was expressed as specific activity (units per mg of protein). One unit of activity represented the amount of enzyme that released one pmole of resorufin in one minute at 37 °C.

#### 5.4.2. CYP2E1

The activity of CYP2E1 was measured according to Zamaratskaia et al. [111], with slight modifications. Briefly, 500 μg microsomal protein was preincubated with 0.2 mM *p*-nitrophenol (Sigma, Bellefonte, PA, USA, 48549) in 100 mM K_2_HPO_4_/KH_2_PO_4_ buffer (pH 6.8) in a final volume of 475 μL at 37 °C for 5 min. The reaction was started by adding 25 μL of 20 mM NADPH (final concentration of NADPH: 1 mM), incubated at 37 °C for 120 min, and immediately, a volume of 20 µL was injected into the HPLC system. The production of *p*-nitrocathechol was analyzed by High-Performance Liquid Chromatography with Diode-Array Detection (HPLC-DAD), using a High-Performance Liquid Chromatography Systems L-3000 from RIGOL Technologies, Inc. (Beijing, China), equipped with a Kinetex EVO C18 column (150 *×* 4.6 mm, 5 µm). The mobile phase consisted of 0.1% trifluoroacetic acid in water as solvent A and 0.1% trifluoroacetic acid in acetonitrile as solvent B. The gradient profile was as follows: 0–10 min 85% solvent A; 10–12 min 85% solvent B; 12–15 min 100% solvent A. The flow rate of the mobile phase was 1 mL/min, and the UV detector was set to 345 nm (Supplementary Materials Figure S2B). For quantification of produced metabolite, *p*-nitrocatechol, a standard calibration curve of *p*-nitrocatechol (Sigma, Saint Louis, MO, USA, N15553-1G) from5 to 400 μM was prepared (Supplementary Materials Figure S2A). One unit of CYP2E1 activity represented the amount of enzyme that produces one pmole of *p*-nitrocatechol in one minute at 37 °C. The Enzyme activity was calculated by relating the amount of produced *p*-nitrocatechol/minute/mg protein to blank incubations and was expressed as specific activity (units per mg of protein).

#### 5.4.3. CYP3A29

The activity of CYP3A29 was measured using the specific substrate nifedipine, as previously described by Sohl et al. [112] and Cheng et al. [113], with the following modifications: 500 μg microsomal protein and 200 μM nifedipine were preincubated in 100 mM K_2_HPO_4_/KH_2_PO_4_ buffer (pH 7.85) in a final volume of 425 μL at 37 °C for 3–5 min. The enzymatic reactions were initiated by addition of the 75 μL NADPH-generating system (50 parts 100 mM glucose 6-phosphate with 25 parts of NADP+ 10 mg/mL and with 1 part of glucose 6-phosphate dehydrogenase from *Leuconostoc mesenteriodes* at 1 mg/mL). After incubation at 37 °C for 10 min, the reaction was terminated by addition of 1.5 mL of ice-cold acetonitrile, followed by centrifugation for 10 min at 15,000× *g* to precipitate the proteins. The supernatants were collected in amber vials and subjected to HPLC analysis, using a High-Performance Liquid Chromatography Systems L-3000 from RIGOL Technologies, Inc. (Beijing, China). The column was Kinetex EVO C18 column (150 *×* 4.6 mm, 5 µm) with the isocratic mobile phase of 0.1% trifluoroacetic acid in water/acetonitrile/methanol (40:30:30) at a flow rate of 1 mL/min at 30 °C. The consumption of nifedipine in the reaction mixture for each of the samples was determined based on calibration curves (Supplementary Materials Figure S3B) constructed from a series of standards of 2.5–250 µM nifedipine (Sigma, Saint Louis, MO, USA, N7634-1G). The remaining substrate was detected as the absorbance at 235 nm (Supplementary Materials Figure S3A). One unit of CYP3A29 activity represented the amount of enzyme that consumes one nmole of nifedipine in one minute at 37 °C. The enzyme activity was calculated by relating the amount of consumed nifedipine/minute/mg protein to blank incubations and was expressed as specific.

#### 5.4.4. GSTA1

Glutathione S-transferase (GST; EC 2.5.1.18) activity was determined by measuring the conjugation rate of GSH with 1-chloro-2,4-dinitrobenzene (CDNB) substrate at 340 nm, according to the method described by Habig et al. [114] and adapted for 96 well plates with a 200 μL final volume per well. One unit of GSTA1 represented the amount of enzyme that releases one µmole of GS-CDNB product in one minute at 25 °C. Enzyme activity was expressed as specific activity (units per mg of protein).

#### 5.4.5. Protein Determination

Each time after thawing a sample aliquot of microsomal fraction for Western blot and enzymatic activity assays, the protein concentration was determined by Bradford method [115] using bovine serum albumin as standard.

### 5.5. Statistical Analysis

Statistical analyses to identify differences in protein expression and enzyme activities were evaluated by one-way ANOVA method performed with GraphPad Prism 3.03 software (GraphPad Software, La Jolla, CA, USA). Post hoc comparisons between all groups were run using the Bonferroni test. The statistical significance (*p* value) was presented for all groups in contrast to the Control group (C). For each analysis, each of biological replicate was run in three technical replicates.

## Data Availability

The data presented in this study are available on request from the corresponding author. The data are not publicly available due to privacy reason.

## Supplementary Materials

The following are available online at https://www.mdpi.com/article/10.3390/toxins13090648/s1, Figure S1: Quantification and separation of resorufin using reversed-phase HPLC (C18). Figure S2: Quantification and separation of 4-nitrocatechol using reversed-phase HPLC (C18), Figure S3: Quantification and separation of nifedipine using reversed-phase HPLC (C18).
