# Supplementary material for: The Reduction of the Combined Effects of Aflatoxin and Ochratoxin A in Piglet Livers and Kidneys by Dietary Antioxidants
Source: Toxins (Basel). 2021 Sep 13;13(9):648. doi: 10.3390/toxins13090648 (PMC8472784; doi:10.3390/toxins13090648)
Supplement: Supplementary file 1 [file toxins-13-00648-s001.zip › toxins-1366525-SI.pdf]

The Reduction of the Combined Effects of Aflatoxin and Ochratoxin A in Piglet Livers and Kidneys by Dietary Antioxidants

Roua Gabriela Popescu, Sorin Avramescu, Daniela Eliza Marin, Ionelia Țăranu, Sergiu Emil Georgescu and Anca Dinischiotu

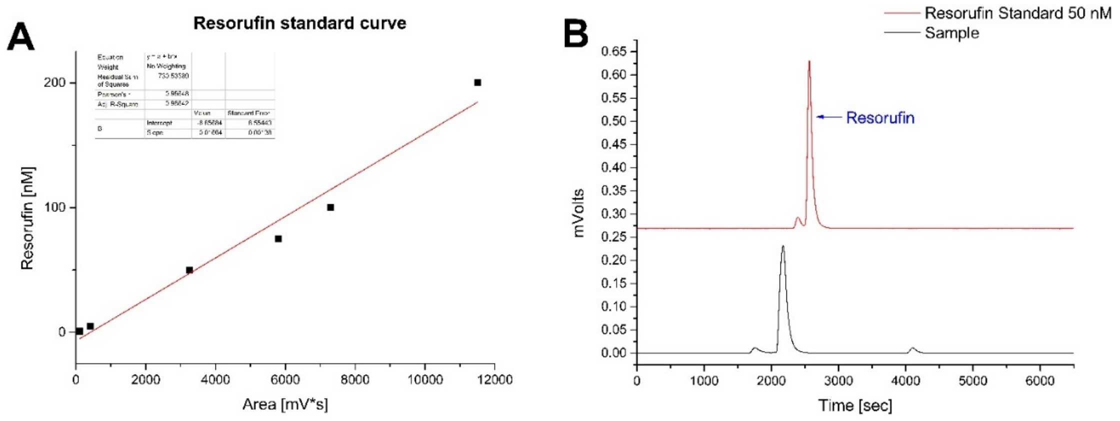

Figure S1. Quantification and separation of resorufin using reversed-phase HPLC (C18). Linear regression with a coefficient of determination of 0.97 was obtained for quantitative determination by integration of the peak area of six resorufin samples with known concentration levels: 1, 5, 50, 75, 100 and 200 nM (**A**). The fluorescence spectra with excitation and emission wavelengths of 560 and 586 nm were recorded using a Varian HPLC system. Mobile phase was 20 mM K_2HPO_4/KH_2PO_4 buffer (pH 6.8), methanol and acetonitrile (30:35:35 v/v) with a flow rate of 0.5 mL /min at 40 °C, the stationary phase was C18, column dimensions: 150 ×4.6 mm; 3 μm particle size. Sample volume was 20 μL of 50 nM resorufin standard, respective sample of enzymatic reactions. Resorufin elutes at 3.66 minutes and is clearly separated from other similar /impurity molecules (**B**). Obtained data were analyzed using OriginPro 9 software.

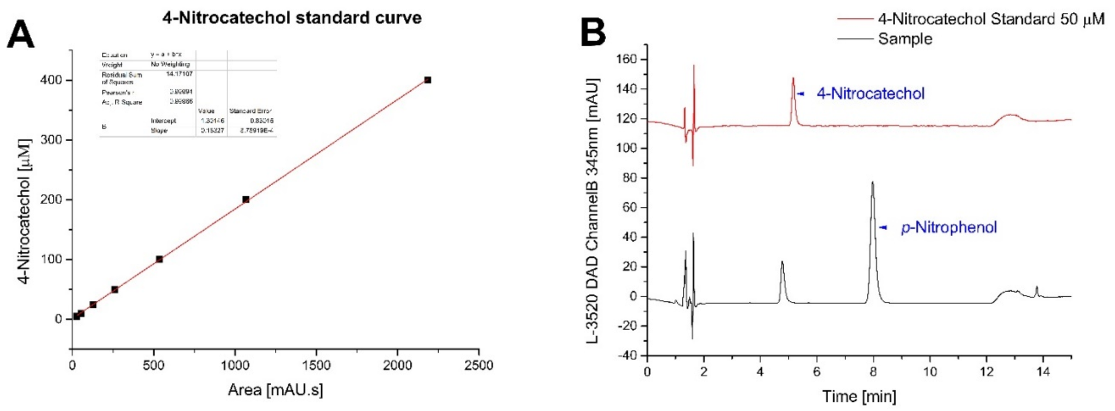

Figure S2. Quantification and separation of 4-nitrocatechol using reversed-phase HPLC (C18). Linear regression with a coefficient of determination of 0.99 was obtained for quantitative determination by integration of the peak area of seven p-nitrocatechol samples with known concentration levels: 5, 10, 25, 50, 100, 200 and 400 μM (**A**). The light absorption spectra at 345 nm were recorded using a High-

Performance Liquid Chromatography Systems L-3000 (RIGOL Technologies, Inc.). Mobile phase was 0.1% trifluoroacetic acid in water as solvent A and 0.1% trifluoroacetic acid in acetonitrile as solvent B. The gradient profile was as follows: 0-10 min 85% solvent A; 10-12 min 85% solvent B; 12-15 min 100% solvent A, with a flow rate of 1 mL /min at 30 °C, the stationary phase was C18, column dimensions: 150 ×4.6 mm; 5 μm particle size. Sample volume was 20 μL of 50 μM p-nitrocatechol standard, respective sample of enzymatic reactions. P-Nitrocatechol elutes at 5.16 minutes and is clearly separated from other similar /impurity molecules (**B**). Obtained data were analyzed using OriginPro 9 software.

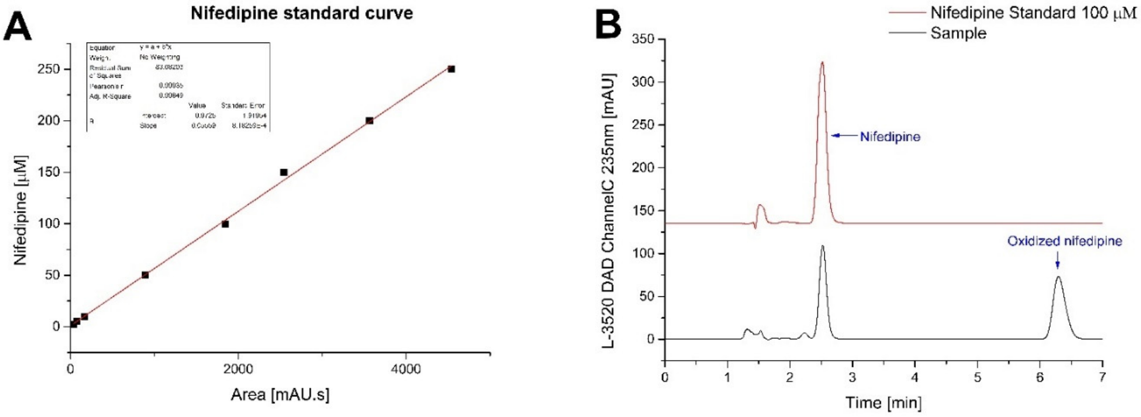

Figure S3. Quantification and separation of nifedipine using reversed-phase HPLC (C18). Linear regression with a coefficient of determination of 0.99 was obtained for quantitative determination by integration of the peak area of six nifedipine samples with known concentration levels: 2.5, 5, 10, 50, 200 and 250 μM (**A**). The light absorption spectra at 235 nm were recorded using a High-Performance Liquid Chromatography Systems L-3000 (RIGOL Technologies, Inc.). Mobile phase was 0.1% trifluoroacetic acid in water /acetonitrile /methanol (40:30:30) with a flow rate of 1 mL /min at 30 °C, the stationary phase was C18, column dimensions: 150 ×4.6 mm; 5 μm particle size. Sample volume was 20 μL of 100 μM nifedipine standard, respective sample of enzymatic reactions. Nifedipine elutes at 2.56 minutes and is clearly separated from other similar /impurity molecules (**B**). Obtained data were analyzed using OriginPro 9 software.
